# Evaluation of the Diagnostic Performance of *Onchocerca volvulus* Linear Epitopes in a Peptide Enzyme-Linked Immunosorbent Assay

**DOI:** 10.4269/ajtmh.17-0756

**Published:** 2018-01-08

**Authors:** Ole Lagatie, Ann Verheyen, Erik Nijs, Bieke Van Dorst, Linda Batsa Debrah, Alex Debrah, Taniawati Supali, Erliyani Sartono, Lieven J. Stuyver

**Affiliations:** 1 Janssen Diagnostics, Janssen R&D, Beerse, Belgium;; 2 Kumasi Centre for Collaborative Research into Tropical medicine, Kwame Nkrumah University of Science and Technology, Kumasi, Ghana;; 3 Faculty of Allied Health Sciences, Kwame Nkrumah University of Science and Technology, Kumasi, Ghana;; 4 Department of Parasitology, Faculty of Medicine, Universitas Indonesia, Jakarta, Indonesia;; 5 Department of Parasitology, Leiden University Medical Center, Leiden, The Netherlands

## Abstract

Diagnostic tools for the detection of infection with *Onchocerca volvulus* are presently limited to microfilaria detection in skin biopsies and serological assessment using the Ov16 immunoglobulin G4 (IgG4) rapid test, both of which have limited sensitivity. We have investigated the diagnostic performance of a peptide enzyme-linked immunosorbent assay (ELISA) based on immunodominant linear epitopes previously discovered. Peptides that were used in these assays were designated *O. volvulus* motif peptides (OvMP): OvMP-1 (VSV-EPVTTQET-VSV), OvMP-2 (VSV-KDGEDK-VSV), OvMP-3 (VSV-QTSNLD-VSV), and the combination of the latter two, OvMP-23 (VSV-KDGEDK-VSV-QTSNLD-VSV). Sensitivity (*O. volvulus* infection), specificity (non-helminth infections), and cross-reactivity (helminth infections) were determined using several panels of clinical plasma isolates. OvMP-1 was found to be very sensitive (100%) and specific (98.7%), but showed substantial cross-reactivity with other helminths. Of the other peptides, OvMP-23 was the most promising peptide with a sensitivity of 92.7%, a specificity of 100%, and a cross-reactivity of 6%. It was also demonstrated that these peptides were immunoreactive to IgG but not IgG4, and there is no correlation with the Ov16 IgG4 status, making them promising candidates to complement this already available test. Combination of the Ov16 IgG4 rapid test and OvMP-23 peptide ELISA led to a sensitivity of 97.3% for the detection of *O. volvulus* infection, without compromising specificity and with minimal impact on cross-reactivity. The available results open the opportunity for a “*clinical utility use case*” discussion for improved *O. volvulus* epidemiological mapping.

## BACKGROUND

One of the 20 communicable diseases listed on the World Health Organization (WHO) list of neglected tropical diseases is onchocerciasis (or river blindness), which is caused by infection with the filarial nematode *Onchocerca volvulus*.^1–3^ With at least 120 million people at risk to be infected, the majority of people suffering from this parasitic disease live in Africa.^4,5^ Recent updates on the Americas have reported that most of the last known areas of *O. volvulus* transmission have been freed of the parasite.^6,7^ However, challenges remain in the Amazonian focus straddling Venezuela and Brazil.^8^ In an effort to eliminate this disease, mass drug administration (MDA) programs with ivermectin have been ongoing for almost three decades.^9,10^ In 2016, the WHO released updated criteria for stopping MDA as a result of transmission interruption.^11^ The technical procedures and the corresponding cutoff values to confirm transmission interruption included the following: 1) screening pools of black flies by the polymerase chain reaction (PCR) for the DNA repeat sequence Ov150; minimal elimination value is < 1/2,000 Ov150-positive flies and 2) serological screening of school-aged children < 10 years of age for immunoglobulin G4 (IgG4) antibodies to the Ov16 antigen; elimination value is antibody prevalence < 0.1%. For the latter, a rapid-format test for the detection of IgG4 antibodies to the parasitic Ov16 antigen is available.^12–17^

We have previously performed a proteome-wide scanning of the *O. volvulus* proteome for the presence of linear epitopes.^18^ Of the list of 249 peptides that were found to contain immunodominant epitopes, more than half of them appeared to contain one of three epitope-forming motifs ^1^PxxTQE^6^, ^1^DGxDK^5^, and ^1^Qx(S/T)N(L/I)D^6^. In the study presented here, we have assessed the diagnostic performance of a peptide enzyme-linked immunosorbent assay (ELISA) using peptides containing these minimal epitopes and combinations thereof.

## MATERIALS AND METHODS

### Study samples.

All samples used in this study were de-identified before being provided and usage of these samples for research purposes was approved by an ethical committee or Institutional Review Board.

Plasma samples from *O. volvulus*–infected individuals were collected as part of a field study in Ghana. This study was undertaken in an onchocerciasis-endemic community located in Adansi South District along the Pra river basins in the Ashanti region of Ghana. Physical examinations were performed to identify those subjects having palpable nodules. Skin snips (biopsies) were then taken to determine the microfilarial (mf) load in the skin.^19^ Most subjects were participating in MDA programs. A total of 97 nodule-positive subjects who donated plasma samples were included.

In addition, a second set of 13 samples from *O. volvulus*–infected individuals, collected in Cameroon by Dr. Nutman, was obtained through the Filariasis Research Reagent Resource Center (FR3), Division of Microbiology and Infectious Diseases, National Institute of Allergy and Infectious Diseases (NIAID), National Institutes of Health (NIH). Information on *O. volvulus* infection (number of microfilaria/mg skin and number of palpable nodules) was provided by FR3, along with demographic information (Table 1). All infected individuals had at least two palpable nodules and 25 mf/mg skin (microfilaridermia) as determined by skin snips. Sera were collected from clotted blood obtained by venipuncture.

**Table 1:** Demographic information of study populations used for determination of diagnostic performance

Characteristic	Group										
	*Onchocerca volvulus*–infected		Non-helminth–infected						Helminth-infected		
	*O. volvulus* Cameroon	*O. volvulus* Ghana	HC southern Africa	HC Belgium	HIV	HCV	Dengue	Asthma	*Wb*	*Bm*	STH
Origin	Cameroon	Ghana	Southern Africa	Belgium	USA	USA	Vietnam	USA	Sri Lanka (8)	Indonesia (Central Sulawesi)	Indonesia (Flores)
									Tahiti (2)		
Number of patients	13	97	10	49	25	25	25	25	10	20	20
Age, median (min–max)	54 (20–72)	47 (21–85)	21 (17–47)	40 (23–59)	n.a.	n.a.	26 (4–67)	46 (17–91)	33 (13–48)	19 (10–45)	40 (25–75)
Gender, *n* (%)											
Male	6 (46)	54 (56)	7 (70)	22 (45)	n.a.	n.a.	10 (40)	11 (44)	6 (60)	10 (50)	2 (10)
Female	7 (54)	43 (44)	3 (30)	27 (55)	n.a.	n.a.	15 (60)	14 (56)	3 (30)	10 (50)	18 (90)
Unknown	0 (0)	0 (0)	0 (0)	0 (0)	0 (0)	0 (0)	0 (0)	0 (0)	1 (10)	0 (0)	0 (0)
Source*	FR3	KCCR	TS	Janssen	MC	MC	DLS	BR	FR3	UI	UI
Ov16 IgG4 positive, *n*	12	66	0	0	0	0	0	1	0	0	0

*Bm* = *Brugia malayi*; HC = healthy control; HCV = hepatitis C virus; HIV = human immunodeficiency virus; IgG4 = immunoglobulin G4; n.a. = not available; STH = soil-transmitted helminths; *Wb* = *Wuchereria bancrofti*.

* BR = Bioreclamation; DLS = Discovery Life Sciences; FR3 = Filariasis Research Reagent Resource Center; KCCR = Kumasi Center for Collaborative Research; MC = Mayo Clinic; TS = Tissue Solutions; UI = Universitas Indonesia.

For the non-helminth–infected control samples, demographic information is also provided in Table 1. A first healthy control sample set was composed of 10 serum samples from individuals from Southern Africa, collected in Food and Drug Administration-regulated donor centers in the USA, and was provided by Tissue Solutions Ltd. (Glasgow, Scotland). A second healthy control sample set was composed of 49 plasma samples from healthy individuals from Belgium.^20–24^ Furthermore, different sets of plasma samples were obtained from Discovery Life Sciences, Inc. (Los Osos, CA), Bioreclamation IVT (Baltimore, MD), and FR3 or Mayo Clinic (Jacksonville, FL).

For the cross-reactivity panels (non-*Onchocerca* helminth–infected individuals), a set of 10 samples from *Wuchereria bancrofti*–infected individuals, collected in Tahiti by Dr. Perolat or Sri Lanka (unknown collector), was obtained through the FR3, Division of Microbiology and Infectious Diseases, NIAID, NIH. Information on *W. bancrofti* infection (number of microfilaria/mL) was provided by FR3, along with demographic information (Table 1). Also, two sets of samples were collected in Indonesia. A first set was collected from individuals with *Brugia malayi* infection in central Sulawesi; a second set was collected in a region highly endemic for soil-transmitted helminths (STH) in Flores Island (Table 1).

Two additional groups were also included for which the helminth infection status is unknown. A first set of samples was collected from malaria-infected individuals from rural areas in Vietnam, which is endemic for STH and lymphatic filariasis, mainly *B. malayi*.^25–29^ Samples were provided by Discovery Life Sciences, Inc. A second set of samples was obtained from individuals in France (travelers and immigrants) who presented with symptoms of filariasis and who had a positive serology test against *Acanthocheilonema viteae* somatic antigens, which is used for screening of suspected clinical cases of human filarial infections (including *W. bancrofti*, *B. malayi*, *Loa loa*, *O. volvulus*, and *Mansonella perstans*). Samples were provided by Tissue Solutions Ltd. An overview of the patient demographics of both panels is provided in Table 2.

**Table 2:** Demographic information of study populations with unknown helminth infection status

Characteristic	Group	
	Malaria	Filariasis
Origin	Vietnam	France
No. of patients	25	17
Age, median (min–max)	22 (18–40)	44 (15–76)
Gender, *n* (%)		
Male	24 (96)	10 (59)
Female	1 (4)	6 (35)
Unknown	0 (0)	1 (6)
Source*	DLS	TS
Ov16 IgG4 positive, *n*	0	0

IgG4 = immunoglobulin G4.

* DLS = Discovery Life Sciences; TS = Tissue Solutions.

### Total IgG peptide ELISA.

Carboxy-terminally biotinylated synthetic peptides were synthesized by following standard procedures and purchased from PEPperPRINT GmbH (Heidelberg, Germany). For the determination of peptide-specific serum antibody levels, a peptide ELISA was developed and set up as follows. Streptavidin-Coated high capacity plates (Thermo Fisher Scientific, Breda, The Netherlands) were rinsed once with 200 μL phosphate buffered saline (PBS) + 0.05% Tween-20 (washing buffer). The plates were incubated with continuous shaking for 1 hour at room temperature with 100 μL of the selected biotinylated peptides, which were diluted at 1 μg/mL in PBS. In the “no peptide” control wells and positive control wells, PBS was added instead. The plates were rinsed three times with washing buffer. Then, the different wells were covered with 100 μL of human serum samples, diluted 200-fold in SuperBlock^™^ blocking buffer (Thermo Fisher Scientific). In “blank” control wells, SuperBlock blocking buffer was added instead, and in positive control wells, 6.25 ng/mL biotinylated Horse Radish Peroxidase in SuperBlock blocking buffer was added. The plate was incubated at room temperature for 1 hour. After incubation, a 5-fold rinsing cycle with washing buffer was performed. Then, the secondary antibody solution was added to each well. The solution contained an affinity-purified Donkey antihuman IgG (H+L) peroxidase conjugate (Jackson Immuno Research Europe Ltd., Newmarket, United Kingdom) diluted 1:10,000 in blocking solution. The reaction mixture was incubated at room temperature for 30 minutes. At the end of the incubation period, the plates were rinsed five times with washing buffer and treated with 100 μL 1-Step^™^ Ultra TMB-ELISA substrate solution (Thermo Fisher Scientific). After 10 minutes of incubation, the colorimetric reaction was stopped with 100 μL 1 N HCl. The plate was then read by using the SpectraMax Plus 384 microplate reader (Molecular Devices, Sunnyvale, CA) at a wavelength (λ) of 450 nm. For each sample, peptide-specific signals were corrected for the “no peptide” control signals to obtain the background-corrected optical density values. In cases where the peptide-specific signal was lower than the background, the result was adjusted to “0.”

### IgG4 peptide ELISA.

Peptide ELISAs were performed as described earlier, except for the secondary antibody used, which was a mouse monoclonal HP6025 antihuman IgG4 (HRP) from Abcam (Cambridge, United Kingdom) and used after diluting 1:10,000 in blocking solution.

### Onchocerciasis Ov16 IgG4 rapid test.

The presence of IgG4 antibodies against the *O. volvulus* antigen Ov16 was determined using the SD BIOLINE Onchocerciasis IgG4 test (Standard Diagnostics, Gyeonggi-do, Republic of Korea), according to the manufacturer’s instructions. Briefly, 10 μL of plasma was added to the round sample well on the lateral flow strip, immediately followed by the addition of four drops of assay diluent into the square assay diluent well. After 1 hour, the tests were scored. The tests were considered positive only when both the test and the control line were visible. Faint lines were also considered positive, as recommended by the manufacturer.

### Onchocerciasis-selected peptides.

Based on the previous discovery of three immunodominant motifs and the epitope mapping results for these motifs, peptides were designed that contain the minimal epitope flanked both N- and C-terminally by a simple Val-Ser-Val linker (Table 3).^18^ For the nonessential amino acid residues in the epitope sequence, amino acid residues were chosen that showed optimal response in the epitope mapping experiments. Consequently, the epitope sequences used in the peptides were not present as such in any *O. volvulus* protein, but represented the optimized consensus epitope sequences of a set of cross-reacting epitopes. All peptides were designated *O. volvulus* motif peptides (for OvMP).

**Table 3:** Peptides used in this study

Peptide name	Sequence
OvMP-1	VSV-EPVTTQET-VSV-Biotin
OvMP-2	VSV-KDGEDK-VSV-Biotin
OvMP-3	VSV-QTSNLD-VSV-Biotin
OvMP-23	VSV-KDGEDK-VSV-QTSNLD-VSV-Biotin

OvMP = *Onchocerca volvulus* motif peptides.

### Statistical analysis.

For each peptide investigated, receiver operating characteristic (ROC) analysis was performed. Several sample sets from non-helminth–infected individuals were used as a control group (to determine specificity). These included healthy controls from both Belgium and South Africa, human immunodeficiency virus–infected or hepatitis C virus–infected individuals from the USA, dengue-infected individuals from Vietnam, and asthma patients from the USA. Two groups of confirmed (microfilaria positive, PCR positive, nodule positive, or Ov16 IgG4 positive) *O. volvulus*–infected individuals were used as the positive group (to determine sensitivity): one from Cameroon and one from Ghana. ROC analysis was performed using all non-helminth controls versus all *O. volvulus* positive samples and cutoffs were determined as the point with maximal Youden’s index ([Sensitivity + Specificity] − 1). Based on these cutoffs, sensitivity and specificity of each peptide ELISA were determined, as well as cross-reactivity with other helminths. This cross-reactivity was determined on sample sets from *W. bancrofti*-, *B. malayi*- and STH-infected individuals. All analyses were performed using GraphPad Prism 7 (GraphPad Software, Inc., La Jolla, CA). In the case of OvMP-23, the cutoff was defined as the point with maximal specificity, at the expense of a slight decrease of sensitivity.

For comparison of different groups, a two-tailed Mann–Whitney test was performed using GraphPad Prism 7. *P* value < 0.05 was considered significantly different.

## RESULTS AND DISCUSSION

### IgG antibodies against peptides with immunodominant motifs.

**Figure 1.**
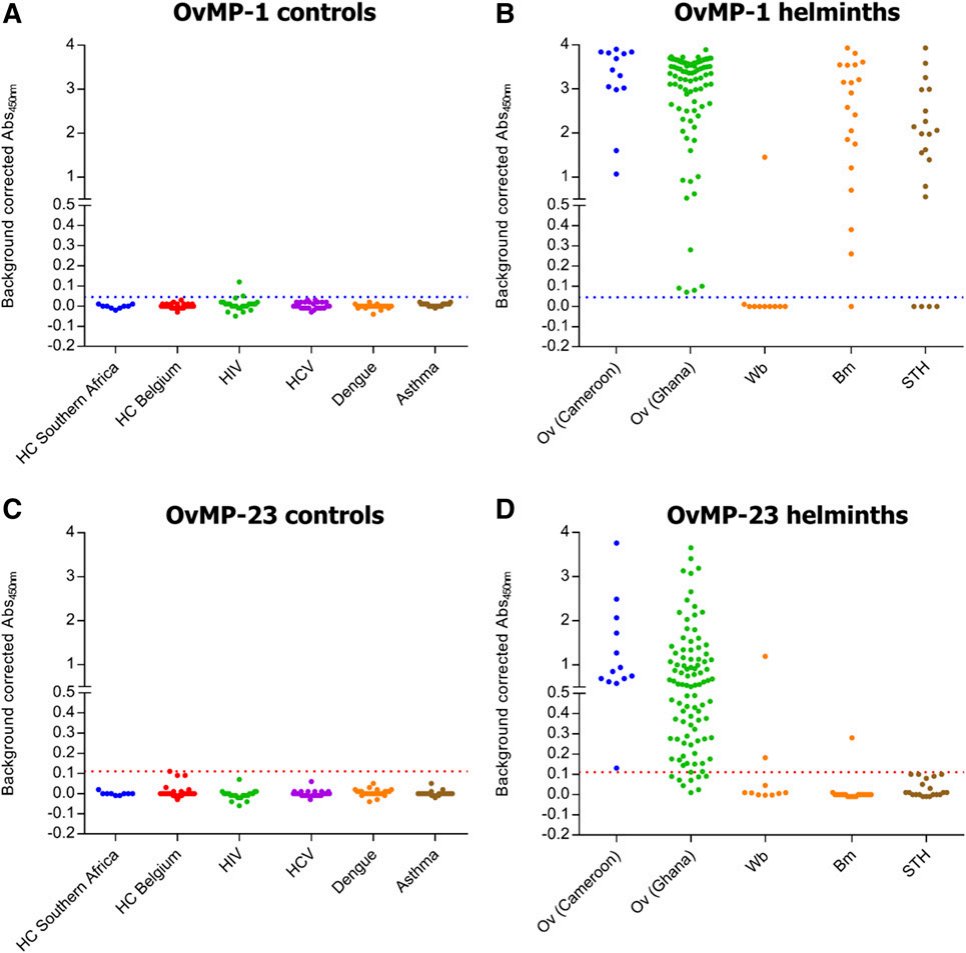
Assessment of immune response against OvMP-1 and OvMP-23. Immunoreactivity against OvMP-1 (**A**, **B**) and OvMP-23 (**C**, **D**) was determined in control groups (**A**, **C**) and in helminth-infected individuals (**B**, **D**). Dotted lines indicate the cutoff determined by receiver operating characteristic analysis (blue for OvMP-1, red for OvMP-23). All samples used in this graph are described in Table 1. OvMP = *Onchocerca volvulus* motif peptides. This figure appears in color at www.ajtmh.org.

The peptides containing consensus immunodominant linear epitope motifs from *O. volvulus* were used to set up peptide ELISA and the diagnostic performance of these peptides was assessed. A total of 110 samples from *O. volvulus*–infected individuals were used to determine sensitivity, whereas 159 samples from non-helminth–infected individuals were used for the determination of specificity. To assess cross-reactivity with other helminths, 50 samples from *W. bancrofti*, *B. malayi*, or STH-infected individuals were used. Table 4 summarizes the performance characteristics of the three peptides that were investigated in this study. As an example, individual data of all OvMP-1 data are presented in Figure 1A and B.

**Table 4:** Performance characteristics of *Onchocerca volvulus* minimal epitope containing peptides

	OvMP-1	OvMP-2	OvMP-3	OvMP-23
Cut-off*	0.045	0.025	0.015	0.11
Sensitivity (%)	100.0	99.1	89.1	92.7
Specificity (%)	98.7	95.6	88.6	100.0
Cross-reactivity (%)	72.0	20.0	10.0	6.0

OvMP = *O. volvulus* motif peptides.

* Background-corrected absorbance.

The peptide OvMP-1 appears to be very specific (98.7%) and to give a positive response in all (100%) *O. volvulus*–infected individuals. However, also in most other helminth-infected individuals, a positive response is observed (72%). This high cross-reactivity is mostly apparent for *B. malayi* and STH, while only a few samples from *W. bancrofti*–infected individuals appear to react with this peptide.

The peptides OvMP-2 and OvMP-3 showed sensitivities of 99.1% and 89.1%, respectively. The specificity of the assays was 95.6% and 88.6% for OvMP-2 and OvMP-3, respectively. A substantial cross-reactivity with other helminths of 20% was observed for OvMP-2, whereas for peptide OvMP-3 this was 10%.

### Combination of epitopes in a single peptide results in improved diagnostic characteristics.

To improve further on sensitivity and specificity, a combined epitope peptide was constructed where the motif sequences from OvMP-2 and OvMP-3 were linked to each other, separated by a VSV linker. This peptide (called OvMP-23) was also used in a peptide ELISA and its performance characteristics were determined, similarly as described earlier (Table 4, Figure 1C and D). As specificity and minimal cross-reactivity are of high importance, a cutoff has been defined for this peptide that corresponds to 100% specificity. This combined epitope peptide appears to have superior sensitivity and specificity characteristics compared with the individual motifs (92.7% and 100%, respectively) and only 6% of the 50 non-*Onchocerca*–, but helminth-infected individuals were seropositive for this peptide. The data presented here clearly demonstrate that combining two linear epitopes is a successful approach to optimize the sensitivity and specificity of the test.

### IgG4 response against OvMP-1 and OvMP-23.

**Figure 2.**
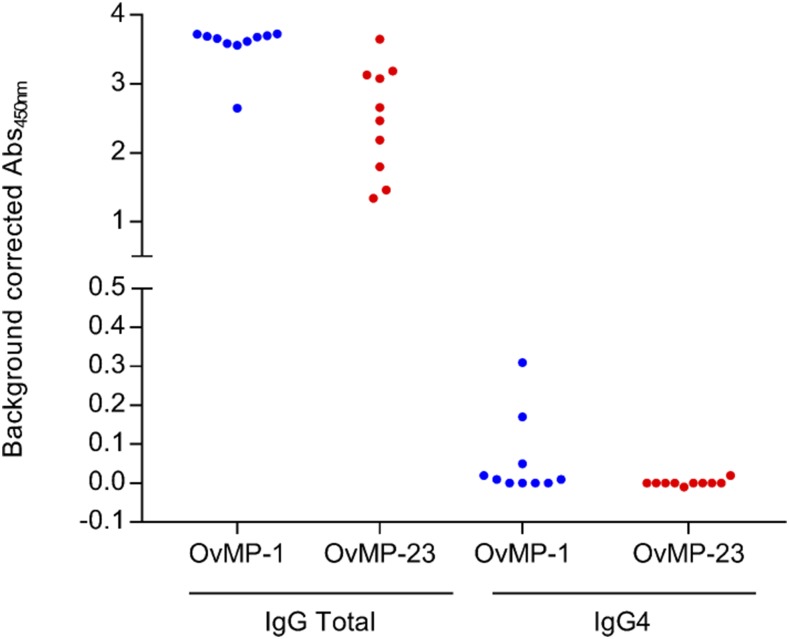
Total IgG and IgG4 isotype response against OvMP-1 and OvMP-23. Immunoreactivity against OvMP-1 (blue) and OvMP-23 (red) was determined in 10 *Onchocerca volvulus*–infected individuals using either total IgG detection antibody or IgG4-specific detection antibody. IgG = immunoglobulin G; OvMP = *O. volvulus* motif peptides. This figure appears in color at www.ajtmh.org.

As it is well-known that the immune response against several parasitic antigens is dominated by an IgG4 response, we have measured IgG4 antibodies against OvMP-1 and OvMP-23 in a selection of samples that have a very strong total IgG response against these peptides (Figure 2). Of the 10 samples evaluated, only two appeared to have detectable IgG4 levels (i.e., background-corrected absorbance > 0.1) that recognize OvMP-1, whereas for OvMP-23 no positive signals were detected. This result indicates that the response against these peptides is dominated by non-IgG4 responses, as was already observed before.^18^ Here again, IgG1 and IgG3 are considered to be the main isotypes.

### Evaluation of the peptides in sample sets from individuals with unknown helminth infection status.

**Figure 3.**
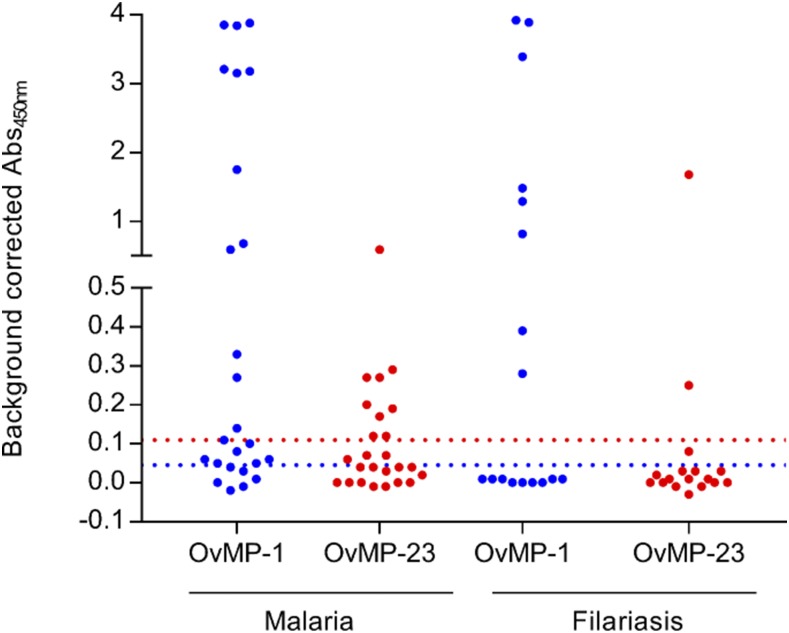
Assessment of immune response against OvMP-1 and OvMP-23 in individuals with unknown helminth infection status. Immunoreactivity against OvMP-1 (blue) and OvMP-23 (red) was determined in malaria-infected individuals from rural Vietnam and in suspected filariasis patients (travelers and immigrants) in France. The blue dotted line indicates the cutoff determined by ROC analysis for OvMP-1, and the red dotted line indicates the cutoff determined by ROC analysis for OvMP-23. All samples used in this graph are described in Table 2. OvMP = *Onchocerca volvulus* motif peptides; ROC = receiver operating characteristic. This figure appears in color at www.ajtmh.org.

In addition to the groups described earlier that had a well-documented helminth infection or exposure status, we also assessed the response against OvMP-1 and OvMP-23 in sample sets with limited or no helminth-related information (Figure 3). In the first set of 25 malaria-infected samples from Vietnam, 19 samples were positive for OvMP-1. As *O. volvulus* is not endemic in Vietnam, exposure to or infection with another infectious agent must be responsible for this immune response. Given the high prevalence of *B. malayi* and STH in this country and the fact that we showed earlier that *B. malayi* and/or STH clearly cross-react with OvMP-1, it is reasonable to assume that infection with or exposure to *B. malayi* or STH is responsible for the observed reactivity. However, as *Plasmodium falciparum*, the causative agent of malaria, is known to express a protein called the interspersed repeat antigen that contains highly immunogenic tandem repeat sequences that perfectly match with the motif in OvMP-1, it cannot be excluded that the reactivity toward OvMP-1 is caused by the malaria infection.^30–32^ Also, nine of these samples tested positive for OvMP-23, which might potentially be caused by cross-reactivity with *B. malayi* or exposure to other filaria species. At this point, it is not clear what is causing the reactivity of these samples to this peptide.

In the second set of 17 filariasis patients, eight were positive for OvMP-1, whereas only two tested positive for OvMP-23. These data suggest that OvMP-1 identifies a proportion of helminth (filariasis)-infected individuals. As discussed earlier, OvMP-1 shows some reactivity to *W. bancrofti* infection, which might be reflected in the data of these filariasis samples. OvMP-23, on the other hand, only reacts in a limited number of filariasis patients. Based on the data obtained in the well-characterized sample sets described earlier, we can now state that the nine samples that do not respond to OvMP-1 are most likely from *W. bancrofti*–infected individuals, whereas the six samples that only respond to OvMP-1 are from *B. malayi*–infected individuals and the samples that respond to both peptides are from *O. volvulus*–infected individuals. However, as no specific information is available for these individuals, we are unable to confirm these conclusions.

### IgG antibodies against peptides are not related to Ov16 IgG4 status.

**Figure 4.**
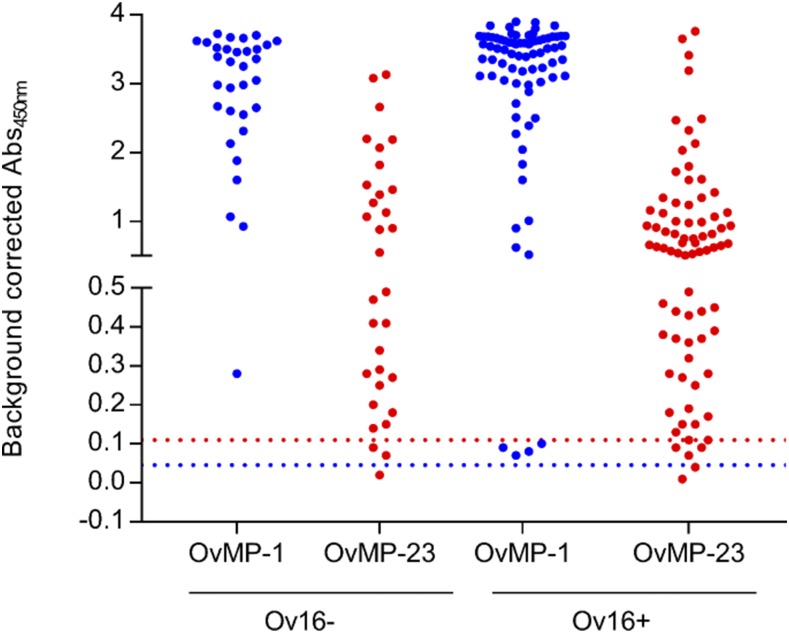
Immune response against OvMP-1 and OvMP-23 according to Ov16 IgG4 status. Immunoreactivity against OvMP-1 (blue) and OvMP-23 (red) was determined in *Onchocerca volvulus*–infected individuals who were grouped according to their Ov16 IgG4 status. The blue dotted line indicates the cutoff determined by ROC analysis for OvMP-1, and the red dotted line indicates the cutoff determined by ROC analysis for OvMP-23. IgG4 = immunoglobulin G4; OvMP = *O. volvulus* motif peptides; ROC = receiver operating characteristic. This figure appears in color at www.ajtmh.org.

All samples that were used for the assessment of the diagnostic performance of the different peptides were also tested with the Ov16 IgG4 rapid test. All non-*Onchocerca*–infected samples were Ov16 negative, except for one sample from an asthma patient (from the USA). In the *Onchocerca*-infected individuals, 78 of a total of 110 samples were positive for Ov16 IgG4, corresponding to a sensitivity of 70.9%, which is comparable to previous reports.^12–15,33^ We have grouped samples based on their Ov16 IgG4 status and investigated whether there was a correlation between Ov16 IgG4 and peptide serology (Figure 4). The results clearly demonstrate that peptide serology and Ov16 IgG4 serology can be used as complementary markers for *Onchocerca* infection. In the sample set investigated in this study, 29 of 32 Ov16 negative samples had antibodies against OvMP-23, resulting in a total sensitivity of 97.3% for the combined Ov16 and peptide serology test. This is also reflected in the fact that for both OvMP-1 and OvMP-23, there is no statistical difference between the Ov16-positive and Ov16-negative groups (*P* value > 0.05).

## CONCLUSION

We have developed peptide ELISAs based on three consensus immunodominant linear epitope motifs from *O. volvulus*. The assay based on OvMP-1 not only has excellent sensitivity for *O. volvulus*, but also cross-reacts substantially with other helminths. Further investigation using clinical samples from well-characterized helminth-infected and/or exposed individuals will be needed to have a clear picture on which helminths cross-react in this assay. The assay based on OvMP-23 has been shown to have a specificity of 100% with a sensitivity of 92% and minimal cross-reactivity with other helminths. When used in conjunction with the Ov16 IgG4 test, a total sensitivity of 97.3% was obtained. The available results open the opportunity for a “clinical utility use case” discussion for improved *O. volvulus* epidemiological mapping.
